# Preexisting Oral Anticoagulant Therapy Ameliorates Prognosis in Hospitalized COVID-19 Patients

**DOI:** 10.3389/fcvm.2021.633878

**Published:** 2021-05-13

**Authors:** Guido Iaccarino, Guido Grassi, Claudio Borghi, Davide Grassi, Costantino Mancusi, Maria Lorenza Muiesan, Massimo Salvetti, Massimo Volpe, Claudio Ferri

**Affiliations:** ^1^Department of Advanced Biomedical Sciences, Federico II University, Naples, Italy; ^2^Department of Medicine and Surgery, University of Milano-Bicocca, Milan, Italy; ^3^Department of Medicine and Surgery Sciences, Alma Mater Studiorum University of Bologna, Bologna, Italy; ^4^Department of Clinical Medicine, Public Health, Life and Environment Sciences, University of L'Aquila, L'Aquila, Italy; ^5^Department of Clinical and Experimental Sciences, University of Brescia, Medicina 2, ASST Spedali Civili Brescia, Brescia, Italy; ^6^Clinical and Molecular Medicine Department, Sapienza University, Rome, Italy; ^7^Sant'Andrea Hospital, Rome, Italy

**Keywords:** multimorbidity, atrial fibrillation, prophylaxis, death, intensive care admissions, COVID-19 outcomes, hypertension, thrombosys

## Abstract

**Objective:** Altered coagulation parameters in COVID-19 patients is associated with a poor prognosis. We tested whether COVID-19 patients on chronic oral anticoagulants (cOACs) for thromboembolism prophylaxis could receive protection from developing more severe phenotypes of the disease.

**Approach and Results:** We searched the database of the SARS-RAS study (Clinicaltrials.gov: NCT04331574), a cross-sectional observational multicenter nationwide survey in Italy designed by the Italian Society of Hypertension. The database counts 2,377 charts of Italian COVID-19 patients in 26 hospitals. We calculated the Charlson comorbidity index (CCI), which is associated with death in COVID-19 patients. In our population (*n* = 2,377, age 68.2 ± 0.4 years, CCI: 3.04 ± 0.04), we confirm that CCI is associated with increased mortality [OR: 1.756 (1.628-1.894)], admission to intensive care units [ICU; OR: 1.074 (1.017-1.134)], and combined hard events [CHE; OR: 1.277 (1.215-1.342)]. One hundred twenty-five patients were on cOACs (age: 79.3 ± 0.9 years, CCI: 4.35 ± 0.13); despite the higher CCI, cOACs patients presented with a lower risk of admissions to the ICU [OR 0.469 (0.250-0.880)] but not of death [OR: 1.306 (0.78-2.188)] or CHE [OR: 0.843 (0.541-1.312)]. In multivariable logistic regression, cOACs confirmed their protective effect on ICU admission and CHE. The CCI remains the most important risk factor for ICU admission, death, and CHE.

**Conclusions:** Our data support a mechanism for the continuation of cOAC therapy after hospital admission for those patients who are on chronic treatment. Our preliminary results suggest the prophylactic use of direct cOACs in patients with elevated CCI score at the time of the COVID-19 pandemic even in absence of other risks of thromboembolism.

## Introduction

The current epidemic of COVID-19 has put the world population and health care systems under enormous stress, acting as an accelerator for death in the older population and anticipating the failure of hospitalocentric administration of health care in light of the increased request for hospital admissions. The scientific community has to, therefore, identify how to protect the high-risk population from the development of critical conditions that would increase the request for high-intensity care. It is now clear from the available literature that older and multimorbid patients are at risk of worse outcomes of COVID-19 (1, 2). Emerging evidence, though, proposes that the more severe outcomes of COVID-19 are also associated with alteration in coagulation patterns. The evidence that altered coagulation parameters in COVID-19 patients is associated with poor prognosis (3, 4) and has led us to hypothesize that the virus can cause an endothelial disease with systemic manifestation due to increased thrombosis (5). Low-molecular-weight heparin anticoagulation in the intensive care unit is associated with better prognosis in severe COVID-19 patients (6). Indeed, in this scenario, anticoagulant treatments are indicated by the majority as pivotal for the management of COVID-19 (7, 8). We explore the possibility that COVID-19 patients on chronic oral anticoagulants (cOACs) for a concomitant condition (i.e., atrial fibrillation, mechanic valvular replacement, pulmonary thromboembolism prophylaxis) before admission receive protection from more severe outcomes.

## Methods

We designed a cross-sectional, multicenter, observational study involving 26 hospitals approached through the Italian Society of Hypertension network in 14 regions of Italy to achieve a nationally representative population sample (SARS-RAS Study) (9). The study is based on an online questionnaire to be filled in with data collected from the hospital charts of COVID-19 patients (see Supplementary Material). The patient cohort included 2,377 patients aged 18-101 years who were referred to the hospital for symptoms of COVID-19. All patients included in the questionnaire were diagnosed with COVID-19 according to World Health Organization interim guidance (10). The observation period started March 9 and ended May 9, 2020. The study was performed following article 89 of General Data Protection and Regulation (https://gdpr-info.eu). The SARS-RAS study is registered on Clinicaltrials.gov with the accession number NCT04331574. The online questionnaire was distributed among the centers to collect reviewed epidemiological, clinical, and outcomes data from hospital emergency rooms and regular and intensive care wards. Each center designated one or more physicians who were tasked with the acquisition and review of the requested information. Patients were pseudonymized by assigning a deidentified identification code. The questionnaire collected information regarding the center and the age, sex, nationality (Italian or other), and city of origin of the patient. From the anamnesis, we collected whether the patient had a known diagnosis of hypertension, coronary artery disease (history of myocardial infarction, PCI, or CABG), heart failure (based on clinical history), atrial fibrillation, diabetes, chronic kidney disease (anamnestic estimated glomerular filtration rate below 60 ml/min/kg), chronic obstructive pulmonary disease (according to GOLD 2019), obesity (body mass index > 30 kg/m^2^), history of blood and solid tumors, liver disease, or other comorbidities; we annotated the presence of prescribed antihypertensive, anticoagulant, and antidiabetic therapy.

The severity of the disease was classified according to the Chinese Center for Disease Control (11) into three groups: asymptomatic or with light symptoms, moderate symptoms, and severe intensity.

We collected also the outcomes (hospital dismission or exitus). All patients for which the course of the disease was in an active state were classified as such (10).

For each patient, we calculated the Charlson comorbidity index (CCI) based on the available data and according to the original description of the score (12). Descriptive analyses of the variables were expressed as mean and standard errors or frequencies expressed in absolute numbers and percentages. Continuous variables were analyzed by ANOVA; categorical data were compared using the χ^2^ test. Regression analyses, odds ratio, and confidence intervals were tested on the interest variables grouped by outcomes; multivariable regression analyses were performed on the significant and clinically relevant continuous and categorical variables.

## Results

**Figure 1:**
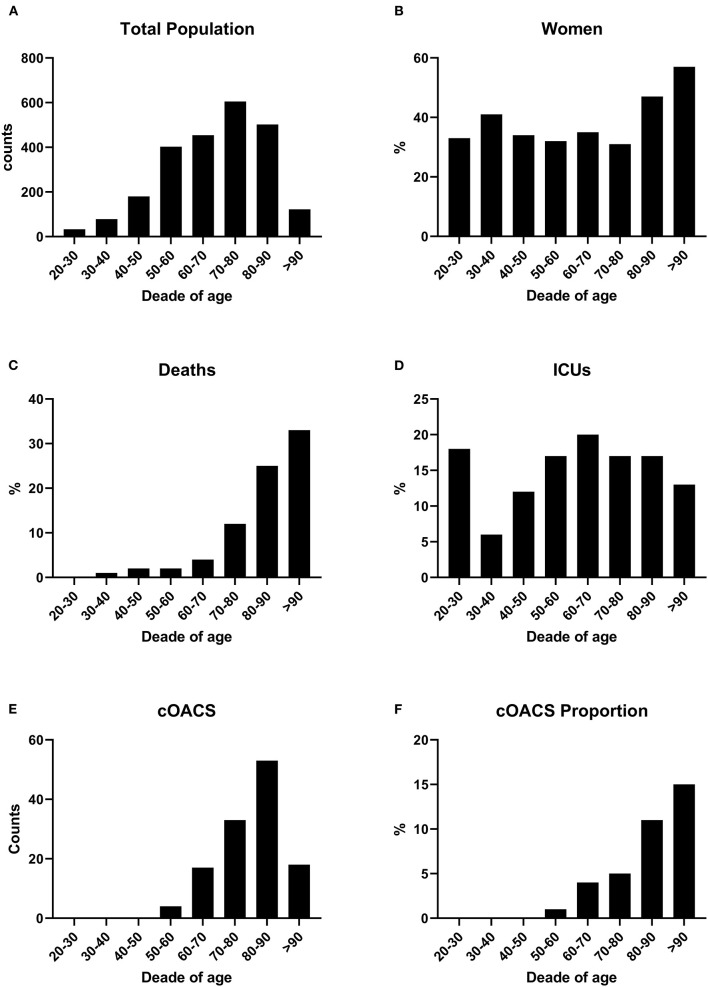
**(A)** COVID-19 patients were grouped by age decades to show the impact of the disease according to age. Patient numbers increased by age decades. **(B)** Women were stably below 40% of total cases up to the age of 80 years. **(C)** Death rates increased with age. **(D)** ICU admissions were stable along all ages. **(E)** cOACs were administered increasingly with age. **(F)** The percentage of patients on cOACs increased with age.

**Figure 2:**
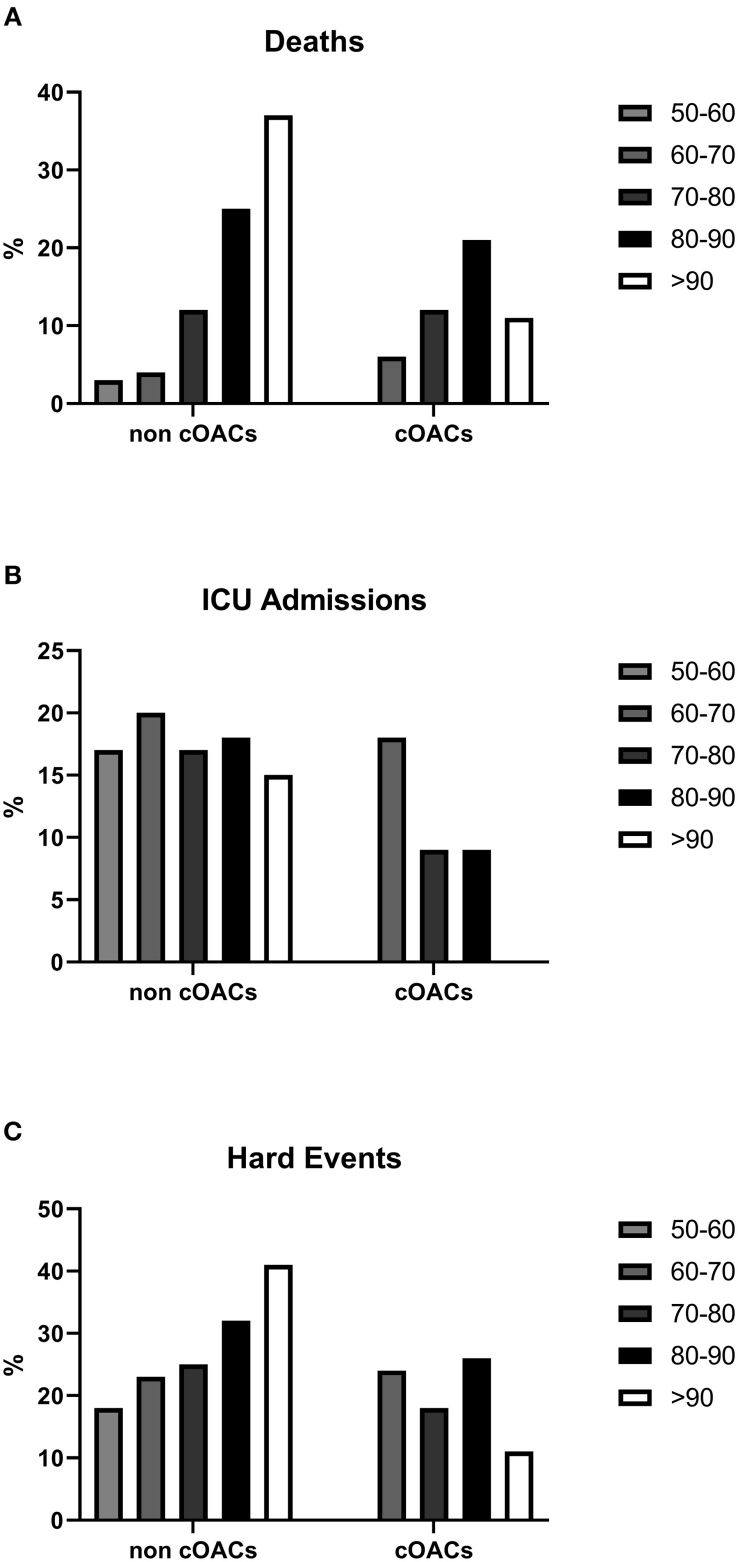
**(A)** Death rates in non-cOACs and cOACs COVID-19 patients. **(B)** ICU admission rates were lower in non-cOACs than cOACs COVID-19 patients. **(C)** CHE (death and ICU admission) rates were similar in non-cOACs and cOACs COVID-19 patients.

**Figure 3:**
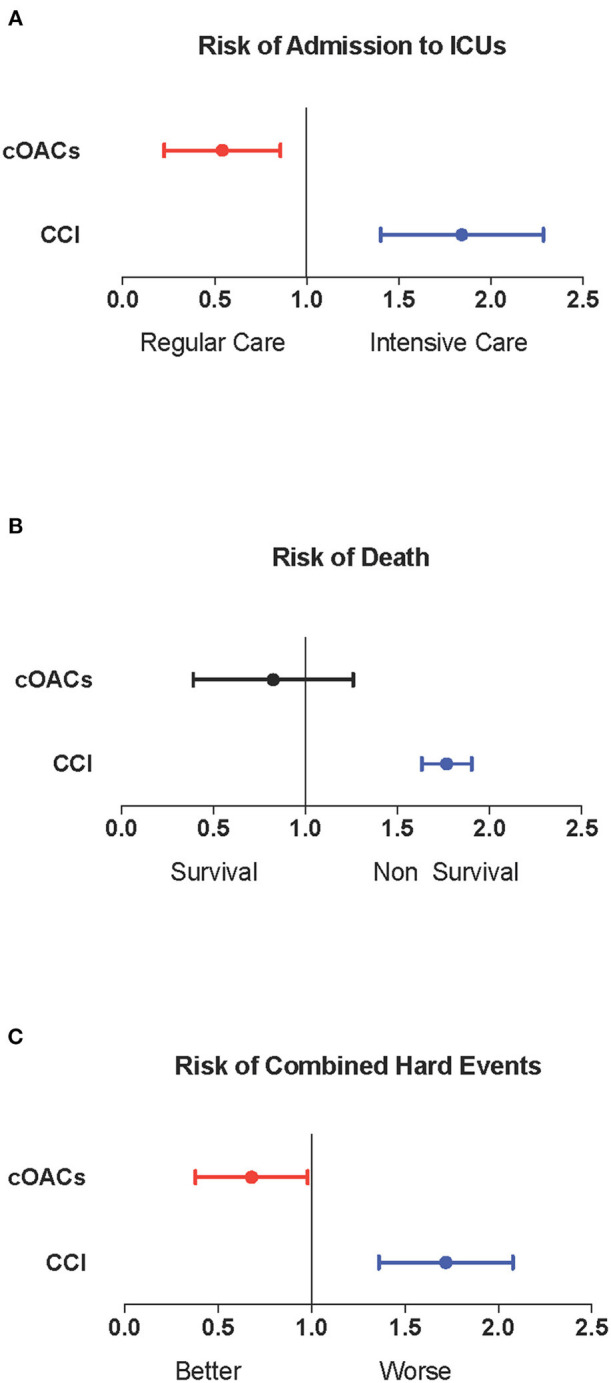
**(A)** Odds ratio and confidence intervals for admission to intensive care units; cOACs: *T* = −0.825, *p* < 0.01; CCI: *T* = 0.085, *p* < 0.003; male sex: *T* = 0.602; *p* < 0.001. **(B)** Odds ratio and confidence intervals for deaths; CCI: *T* = 0.567, *p* < 0.001. **(C)** Odds ratio and confidence intervals for combined hard events (deaths and ICU admissions); cOACs: *T* = −0.455; *p* < 0.05; male sex: *T* = 0.527; *p* < 0.001; CCI:*T* = 0.257; *p* < 0.001. CCI, Charlson comorbidity index; cOACs, chronic oral anticoagulants; ICU, intensive care units.

We collected charts of 2,569 patients. We excluded from the analysis 192 patients for incomplete or discordant data. The analysis was then performed on 2,377 patients. The clinical features of our population are indicated in Table 1. Women were less frequently affected than men, and the mean age indicates that the disease was prevalent among the senior population (Figure 1). We counted 285 deaths and more than 400 patients admitted to intensive care units (ICUs) (Figure 2). We confirm that CCI is associated with increased mortality [Table 2, OR: 1.756 (1.628-1.894)], admission to ICUs [OR: 1.074 (1.017-1.134)], and combined hard events [CHE, OR: 1.277 (1.215-1.342)]. One hundred twenty-five patients were on cOACs for thromboembolic prevention for atrial fibrillation and venous thromboembolism for at least 6 months before the diagnosis of COVID-19. Compared with non-cOACs, cOACs patients were older, included more women, and had a larger CCI (Table 1). Despite the larger CCI, cOACs patients were less likely to be referred to the ICU [Table 2, OR 0.469 (0.250-0.880)], but with a similar risk of death [OR: 1.306 (0.78-2.188)] or CHE [OR: 0.843 (0.541-1.312)]. To ascertain the role of age, multimorbidity (combined in the CCI score), sex, and cOACs on the outcome, we performed a multivariable logistic regression analysis on ICU admission, death, and CHE. cOACs confirmed the protective effect on admission to the ICU and CHE (Figures 3A,C) but not on death (Figure 3B). Sex and CCI remain significant risk factors for ICU access and CHE in COVID-19 patients (Figures 3A-C). In particular, CCI represents the most important risk factor for death in COVID-19 patients.

**Table 1:** Clinical Characteristics of study population.

	**Population**	**cOACs**	**No cOACs**	**Significance**
	**n = 2,377**	**n = 125**	**n = 2,252**	
Age (years)	68.21 ± 0.38	79.35 ± 0.86	67.59 ± 0.39	<0.01
Female	37.3% (888)	45.6% (57)	36.9% (831)	<0.05
CCI	3.04 ± 0,04	4.35 ± 0.13	2.97 ± 0.04	<0.01
Hypertension	59% (1,402)	70.4% (88)	57.86% (1,303)	<0.01
Diabetes	18% (428)	16.8% (21)	18.29% (412)	n.s.
Obesity	7% (166)	4.8% (6)	6.75% (152)	n.s.
BPCO	8% (190)	8% (10)	8.48% (191)	n.s.
CKD	6% (143)	4% (5)	5.68% (128)	n.s.
HF	12% (285)	28.8% (36)	11.14% (251)	<0.01
Death	12% (285)	14.4% (18)	11.41% (257)	n.s.
ICU	17% (404)	8.8% (11)	17.05% (384)	<0.02
Atrial Fibrillation	4.7% (111)	88.8% (111)		
Venous Thrombosis	0.5% (14)	11.2% (14)		

*CCI, Charlson comorbidity index; COPD, chronic obstructive pulmonary disease; CKD, chronic kidney disease; HF, heart failure; ICU, patients in intensive care unit; cOACS, chronic oral anticoagulants. Significance refers to cOACs vs. no cOACS; data are expressed in mean ± SEM or %; in brackets, the absolute numbers; statistical analysis was conducted using ANOVA for continuous variables and chi-squared for distribution*.

**Table 2:** Univariate and multivariate analysis.

	**Wald**	**Sign**.	**Exp(B)**	**95% CI,Lower**	**95% CI, Upper**
**Death, univariate analysis**					
CCI	211.48	**0.00**	1.76	1.63	1.89
Age	123.16	**0.00**	1.07	1.05	1.08
Female sex	0.80	0.37	0.89	0.68	1.15
cOACs	1.03	0.31	1.31	0.78	2.19
**Death, multivariate analysis**					
CCI	212.80	**0.00**	1.76	1.63	1.90
Female sex	1.17	0.28	0.86	0.65	1.14
cOACS	1.08	0.30	1.34	0.77	2.30
**ICU admission, univariate analysis**					
Age	0.64	0.42	1.00	1.00	1.01
Female sex	24.14	**0.00**	0.55	0.43	0.70
CCI	6.50	**0.01**	1.07	1.02	1.13
cOACs	5.56	**0.02**	2.13	1.14	3.99
**ICU admission, multivariate analysis**					
CCI	9.16	**0.00**	1.09	1.03	1.15
Female sex	23.74	**0.00**	0.55	0.43	0.70
cOACs	6.48	**0.01**	2.28	1.21	4.31
**Combined hard events, univariate analysis**					
CCI	92.16	**0.00**	1.28	1.21	1.34
Age	40.27	**0.00**	1.02	1.01	1.03
Female sex	22.33	**0.00**	0.61	0.50	0.75
cOACs	0.57	0.45	1.19	0.76	1.85
**Combined hard events, multivariate analysis**					
CCI	98.16	**0.00**	1.29	1.23	1.36
Female sex	24.21	**0.00**	0.59	0.48	0.73
cOACs	3.86	**0.05**	1.58	1.00	2.48

*CCI, Charlson comorbidity index; cOACS, chronic oral anticoagulants; CI, confidence intervals.*

*Bold p values indicate statistical significance*.

## Discussion

Our study shows that cOACs modify the risk of admission to the ICU and CHE even independently from the major determinants of outcomes in COVID-19, which are age, comorbidity, and sex. This result is well in agreement with the proposed empiric use of anticoagulants for the treatment of severely ill COVID-19 patients (13). So far, this use is based mainly on evidence gathered on a small number of patients of the subgroup analysis from a single retrospective, poorly controlled study (4). The largest number of patients, the multicenter design, and the possibility to perform a multivariable statistic approach confer our study larger statistical soundness.

COVID-19 patients are prone to venous thromboembolism (3) and present with abnormal coagulation parameters, such as D-dimer and APTT (7). The underlying mechanisms include possibly a direct action of the virus on coagulation-competent tissues and cells, such as endothelium (5) and platelets as well as the indirect immune activation and further potentiated hyper-coagulable state, which leads to the development of thromboembolic complications in patients (14). In this scenario, cOACS cannot prevent the infection and the development of COVID-19 but might provide protection toward the consequences of the hypercoagulability state caused by the disease.

Our study also proposes oral anticoagulant therapy as a strategic therapy, in particular, for the early treatment of patients before they become severely ill, as an alternative to the use of parental anticoagulants.

Overall, our data support the proposed use of anticoagulant therapy to prevent a mechanistic approach for the prophylactic use of direct OACs in patients with elevated CCI score at the time of the COVID-19 pandemic to reduce the risk of more severe clinical disease.

In contrast to our findings, a very small study conducted in the United Kingdom indicates a non-significant death reduction in patients treated with either warfarin or DOACs with a paradoxical—although, again, non-significant—increment in ICU admission in patients on OACs (15). Further, progression to an acute respiratory distress syndrome was increased by OACs in 192 hospitalized Italian patients (RR = 1.24, 95% CI 0.56–2.08). However, due to the small sample size, the influence of OACs on disease severity was again non-significant (*p* = 0.465) (16). Thus, our findings seem to represent the only data available in a large population indicating that preexisting OAC therapy can reduce ICU admission in hospitalized patients. Further data from prospective studies could help better our understanding of the prophylactic strategy to choose between different OACs.

## Limitations

The study has a cross-sectional observational design, which could affect the results. For this reason, our research was never intended to be conclusive but rather hypothesis generating. Finally, we cannot discriminate against the role of different anticoagulants because we did not collect the names of the active principles.

## Data Availability Statement

The datasets presented in this study can be found in online repositories. The names of the repository/repositories and accession number(s) can be found below: doi: 10.6084/m9.figshare.12622208.

## Ethics Statement

Ethical approval was not provided for this study on human participants because the study is performed following the article 89 of the General Data Protection and Regulation (https://gdpr-info.eu). Written informed consent for participation was not required for this study in accordance with the national legislation and the institutional requirements.

## The SARS-RAS Investigator group is composed of

Arrigo F. G. Cicero^1^, Claudia Agabiti Rosei^14^, Michele Bevilacqua^10^, Valeria Bisogni^9^, Michele Bombelli^22^, Luca Bulfone^16^, Flaminia Canichella^18^, Giovanni Carpani^22^, Massimo Catanuso^17^, Carmine Savoia^2^, Damiano Rizzoni^15^, Giulia Chiarini^15^, Fernando Chiumiento^21^, Giuseppe Mulè^4^, Rosario Cianci^6^, Giuliano Tocci^2^, Franco Cipollini^23^, Antonio Concistrè^6^, Andrea Dalbeni^10^, Roberto Alberto De Blasi^2^, Riccardo Sarzani^19^, Carolina De Ciuceis^14^, Raffaella Dell'Oro^22^, Antonino Di Guardo^17^, Stefano Perlini^5^, Santo Di Lorenzo^2^, Monica Di Norcia^25^, Giacomo Pucci^9^, Roberto Ervo^11^, Elisabetta Eula^20^, Davide Fabbricatore^14^, Pietro Minuz^10^, Elvira Fanelli^20^, Claudio Letizia^6^, Cristiano Fava^10^, Enzo Grasso^12^, Stefano Carugo^13^, Alessandro Grimaldi^25^, Maddalena Illario^3^, Claudio Invernizzi^22^, Maria Lorenza Muiesan^14^, Elena Iraca^1^, Federica Liegi^1^, Francesca Magalini^26^, Franco Veglio^20^, Paolo Malerba^15^, Leonardo Sechi^16^, Alessandro Maloberti^12^, Costantino Mancusi^3^, Martina Mezzadri^6^, Francesco Fallo^7^, Giulia Molinari^22^, Roberta Mussinelli^5^, Anna Paini^14^, Paola Pellimassi^2^, Paolo Mulatero^20^, Ornella Piazza^8^, Davide Grassi^25^, Luigi Pietramala^6^, Roberto Pontremoli^24^, Fosca Quarti Tevano^22^, Franco Rabbia^20^, Monica Rocco^2^, Anna Sabena^5^, Francesco Salinaro^5^, Paola Schiavi^19^, Maria Chiara Sgariglia^6^, Francesco Spannella^19^, Cristina Giannattasio^12^, Sara Tedeschi^1^, Pierluigi Viale^1^ and the COVID19 Niguarda group.

The SARS-RAS centers are the following:

^1^AO Policlinico Sant'Orsola-Malpighi, Bologna; ^2^AOU Sant'Andrea, Roma; ^3^AOU Federico II, Napoli; ^4^AOU Policlinico Paolo Giaccone, Palermo; ^5^AOU Policlinico San Matteo, Pavia; ^6^AOU Policlinico Umberto I, Roma; ^7^AOU Policlinico Universitario, Padova; ^8^AOU San Giovanni di Dio e Ruggi d'Aragona, PO “Dell'Olmo” Cava de' Tirreni; ^9^AOU Santa Maria, Terni; ^10^AOUI Verona, Italy; ^11^ASL 1 Imperiese, Ventimiglia; ^12^ASST Grande Ospedale Metropolitano Niguarda, Milano; ^13^ASST Santi Paolo e Carlo, Milano; ^14^ASST SPEDALI CIVILI BRESCIA; ^15^ASST SPEDALI CIVILI. PO Montichiari; ^16^ASUI Friuli Centrale, Udine; ^17^Centro Ipertensione Mascalucia, Catania; ^18^INMI Lazzaro Spallanzani, Roma; ^19^INRCA, Ancona, Italy; ^20^Ospedale “Le Molinette”, Torino; ^21^Ospedale di Eboli, Salerno; ^22^Ospedale San Gerardo, Monza; ^23^Ospedale San Jacopo, Pistoia; ^24^Ospedale San Martino, Genova; ^25^PO San Salvatore, L'Aquila; ^26^Ospedale Maggiore, Parma.

## Author Contributions

GI: study design, statistical analysis, and paper writing. GG, CB, MM, and MV: study design and paper editing. DG and MS: data collection and elaboration. CM: data collection and elaboration, statistical analysis, and paper writing. CF: study design and paper writing. All authors contributed to the article and approved the submitted version.

## Conflict of Interest

The authors declare that the research was conducted in the absence of any commercial or financial relationships that could be construed as a potential conflict of interest.
