# Supplementary material for: Preexisting Oral Anticoagulant Therapy Ameliorates Prognosis in Hospitalized COVID-19 Patients
Source: Front Cardiovasc Med. 2021 May 13;8:633878. doi: 10.3389/fcvm.2021.633878 (PMC8155285; doi:10.3389/fcvm.2021.633878)
Supplement: Supplementary file 1 [file Data_Sheet_1.PDF]

Questionario di raccolta dati di associazione tra Ipertensione e severità COVID19

Questo questionario è stato creato dalla Società Italiana dell'Ipertensione Arteriosa per raccogliere dati relativi alla associazione tra assunzione di farmaci antipertensivi inibitori del sistema renina angiotensina e la severità dei sintomi e del quadro clinico della malattia COVID19 in Italia

* Required

1. Email address *

2. Centro di Raccolta dati *

3. Iniziale Paziente *

4. Codice di Riferimento del Paziente (prime tre cifre del centro più le iniziali del paziente) *

5. Età *

6. Sesso *

Mark only one oval.

- Maschio
- Femmina

7. Nazionalità *

Mark only only one oval.

- Italiana
- Altra

8. Città di Origine del paziente *

____

9. Iperteso *

Mark only one oval.

- Si
- No
- Non Nota

10. Cardiopatia Ischemica *

Mark only one oval.

- Si
- No
- Non nota

11. Scompenso Cardiaco *

Mark only one oval.

- Si
- No
- Non nota

12. Diabete Mellito *

Mark only one oval.

- Si
- No
- Non nota

13. Ace Inibitore (i.e. ramipril, lisinopril, zofenopril, etc.) *

Mark only one oval.

- Si
- No
- Non nota

14. AT1 antagonista (i.e. Losartan, candesartan, valsartan, etc.) *

Mark only one oval.

- Si
- No
- Non nota

15. Altro farmaco antipertensivo (beta bloccante, diuretico, calcio antagonista, alfa 1 antagonista) *

Check all that apply.

- [ ] beta bloccante
- [ ] diuretico
- [ ] calcio antagonista
- [ ] alfa 1 Adrenergico bloccante
- [ ] Altro
- [ ] Nessuna

16. Il paziente sta facendo profilassi anti trombotica con anticoagulante orale

Mark only one oval.

- ( ) Option 1

17. Severità COVID-19 *

Mark only one oval.

- ( ) 1 lieve contenuto a domicilio
- ( ) 2 moderata in ricovero ordinario
- ( ) 3 severo in terapia intensiva

18. Esito COVID19 *

Mark only one oval.

- ( ) in fase attiva
- ( ) guarito
- ( ) Exitus

19. Altra condizione *

Mark only one oval.

- BPCO/Asma (incluso tabagismo)
- Malattia genetica
- MAV
- Insufficienza renale (eGFR <60)
- Malattia valvolare
- Miocardite pregressa
- Obesità (BMI>30)
- Fibrillazione atriale
- Nessuna patologia associata
- malattia tiroidea
- Malattia neurologica degenerativa
- Ictus/tia
- ipo/ipertiroidismo
- malattia reumatica o autoimmune
- epatopatia
- dislipidemia
- neoplasia
- leucemia
- deficit cognitivo
- Option 20

20. Se Altro, inserisci una breve descrizione

____

This content is neither created nor endorsed by Google.

Google Forms
